# A typical tuberculosis of the wrist joint and flexor-extensor tendon sheaths: A case report

**DOI:** 10.1097/MD.0000000000047102

**Published:** 2026-01-23

**Authors:** Yuchao Lin, Junlin Liu, Weilong Lin, Yudong Huang, Xiaohua Zheng

**Affiliations:** a Orthopedics Department, Ward 1, Putian 95 Hospital, Putian City, Fujian Province, China; b Orthopedics Department, Hospital of Chengdu Office of The People’s Government of Tibet Autonomous Region, Chengdu City, Sichuan Province, China.

**Keywords:** bone destruction, granulomatous inflammation, misdiagnosis, pigmented villonodular synovitis, tuberculosis, tuberculous tenosynovitis, wrist joint

## Abstract

**Rationale:**

Tuberculous tenosynovitis and arthritis of the wrist are a rare condition that are frequently misdiagnosed due to their nonspecific clinical, serological, and radiological presentations, which often mimics other diseases such as pigmented villonodular synovitis (PVNS). This case highlights the critical diagnostic challenges and the importance of maintaining a high index of suspicion for tuberculosis (TB) in atypical musculoskeletal presentations.

**Patient concerns:**

One patient presented with chronic wrist pain, swelling, and significantly restricted mobility of the wrist and fingers.

**Diagnoses:**

Preoperative TB-specific serological test results (γ-interferon N, P, and T) were negative. Wrist imaging (radiography, computed tomography, and magnetic resonance imaging) revealed synovial hyperplasia, bony destruction, and extensive tenosynovitis affecting both extensor and flexor tendon sheaths beyond the carpal tunnel. Chest computed tomography suggested pneumoconiosis with possible pulmonary TB, leading to a respiratory consultation that could not exclude pulmonary TB infection. The leading preoperative diagnosis was PVNS. Intraoperative frozen section during initial arthroscopy also supported PVNS. However, open surgery discovered distal radioulnar joint instability and extensive bone destruction, which were inconsistent with PVNS. The definitive diagnosis of tuberculous granulomatous inflammation was confirmed postoperatively by histopathological examination of paraffin-embedded sections.

**Interventions:**

The patient underwent initial arthroscopic exploration followed by open surgery. Postoperatively, the patient was received standard quadruple anti-TB drug therapy (isoniazid, rifampicin, pyrazinamide, and ethambutol).

**Outcomes:**

During follow-up, the patient demonstrated significant symptomatic relief and functional improvement after surgical intervention and the initiation of anti-TB chemotherapy.

**Lessons:**

This case underscores that wrist TB can closely mimic PVNS in terms of clinical, radiological, and even intraoperative frozen section findings. Key factors that should raise suspicion for TB include preoperative imaging showing bone destruction, the presence of concurrent pulmonary TB lesions, and intraoperative findings of severe bone or joint damage that are atypical for PVNS. Definitive diagnosis depends on comprehensive histopathological examination. Maintaining a high clinical suspicion is essential to avoid misdiagnosis and ensure appropriate treatment.

## 1. Introduction

Despite significant advances in the diagnosis and treatment of tuberculosis (TB), it remains a major global public health challenge and is one of the top 10 causes of death globally.^[1]^ This is the case even though the incidence of TB decreased by 42.2% between 2010 and 2023.^[2]^ Osteoarticular TB accounts for 1% to 3% of all extrapulmonary TB cases, with wrist joint involvement being particularly rare, representing only 1% of osteoarticular TB cases. It frequently affects the wrist joint and flexor tendons.^[3,4]^ Characterized by an insidious onset, its clinical manifestations typically include joint swelling, pain, and restricted movement, often without the typical constitutional symptoms of TB. Routine screening tests are frequently negative, leading to misdiagnosis or confusion with conditions such as rheumatoid arthritis, nonspecific wrist synovitis, or pigmented villonodular synovitis (PVNS).^[5,6]^ We report a case initially diagnosed pre- and intraoperatively (via frozen section) as PVNS but ultimately confirmed as tuberculous tenosynovitis and arthritis of the wrist. This report discusses the clinical, radiological, and pathological characteristics; diagnostic pitfalls; and treatment strategies.

## 2. Case presentation

A 67-year-old male miner presented with a 6-month history of a progressively enlarging mass, pain, and restricted movement in his right wrist. He denied typical TB symptoms, such as prolonged low-grade fever, fatigue, night sweats, or recent significant weight loss. His body mass index was 17.6 kg/m². He had a history of pneumoconiosis, but denied any known TB contact or residence in endemic areas. The patient denied any history of wrist trauma or surgery. When the pain was severe, oral non-steroidal anti-inflammatory drugs were required for analgesia, and no injection therapy was administered to the wrist.

Physical examination revealed diffuse swelling on the volar and dorsal aspects of the right wrist without erythema. Skin temperature was comparable to that on the contralateral side. The skin of the right finger appeared dusky, with areas of pallor. Palpable masses that were soft and immobile, were noted at the volar midline and ulnodorsal aspects of the wrist. Mild tenderness was observed upon palpation. The distal radioulnar joint (DRUJ) exhibited a positive piano-key sign. Longitudinal compression pain was absent. Significant muscle atrophy was observed on the right forearm. Sensation was diminished in the right thumb, index finger, middle finger, ring finger, and web space compared to that in the left. Active flexion and extension of the right wrist and fingers were not observed. The right wrist was fixed with 10° palmar flexion deformity. Active radial and ulnar deviations of the wrist were restricted. Using the neutral-0 method, the right forearm pronation/supination range was 20°/0°/0°, respectively. The distal perfusion in the right hand was adequate.

### 2.1. Laboratory results

Complete blood count: hemoglobin 113 g(L); white blood cell counts and neutrophil count within normal limits; inflammatory markers: C-reactive protein 5 mg/L; erythrocyte sedimentation rate (ESR) 25 mm/h. T-SPOT.TB (γ-interferon release assay): nil control (N): 0.501 IU/mL (normal), mitogen control (P): >10 IU/mL (normal, indicates valid test), TB antigen (T): 4.506 IU/mL (normal threshold typically >0.35 IU/mL indicates positive in context of valid controls), interpretation: positive for TB antigen response (*T* value > 0.35 IU/mL with valid P and N controls). All other laboratory findings were unremarkable.

### 2.2. Preliminary diagnosis and treatment plan

Based on the chronic clinical course, imaging findings (magnetic resonance imaging, synovial hyperplasia, x-ray/computed tomography [CT], osteolytic destruction, chest CT, cavitary lesions) and laboratory results (unremarkable inflammatory markers; T-SPOT. TB: nil control 0.501 IU/mL, mitogen control > 10 IU/mL, TB antigen 4.506 IU/mL (positive per manufacturer threshold > 0.35 IU/mL), the provisional diagnosis favored PVNS, although tuberculous arthritis remained a differential consideration. Given the extensive disease involvement, joint deformity, and functional impairment, surgical management was indicated wrist arthroscopy-assisted synovial biopsy and synovectomy, with possible conversion to open debridement and arthrodesis.

**Figure 1.**
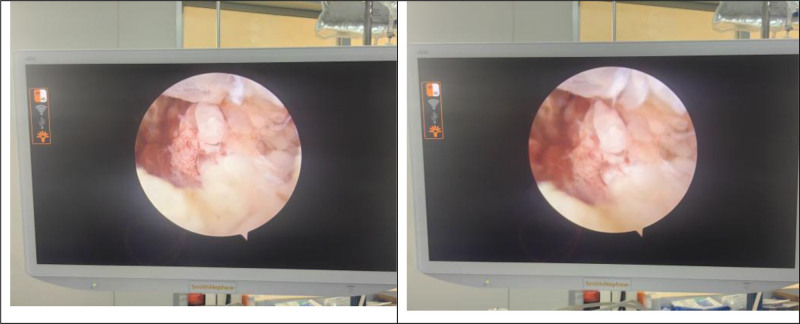
Intraoperative arthroscopic view: no hemosiderin deposition observed.

**Figure 2.**
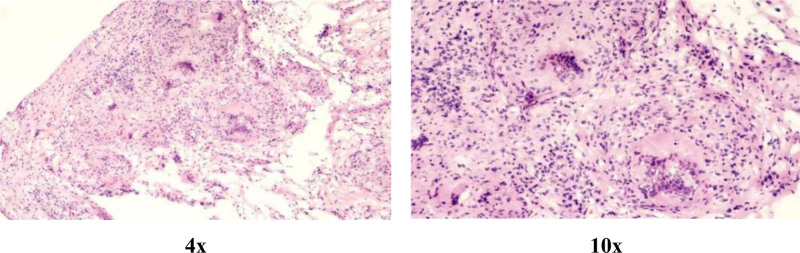
H&E-stained intraoperative frozen sections: ×4 magnification and ×10 magnification. The sections showed only giant cells and synovial cells. H&E = hematoxylin and eosin.

**Figure 3.**
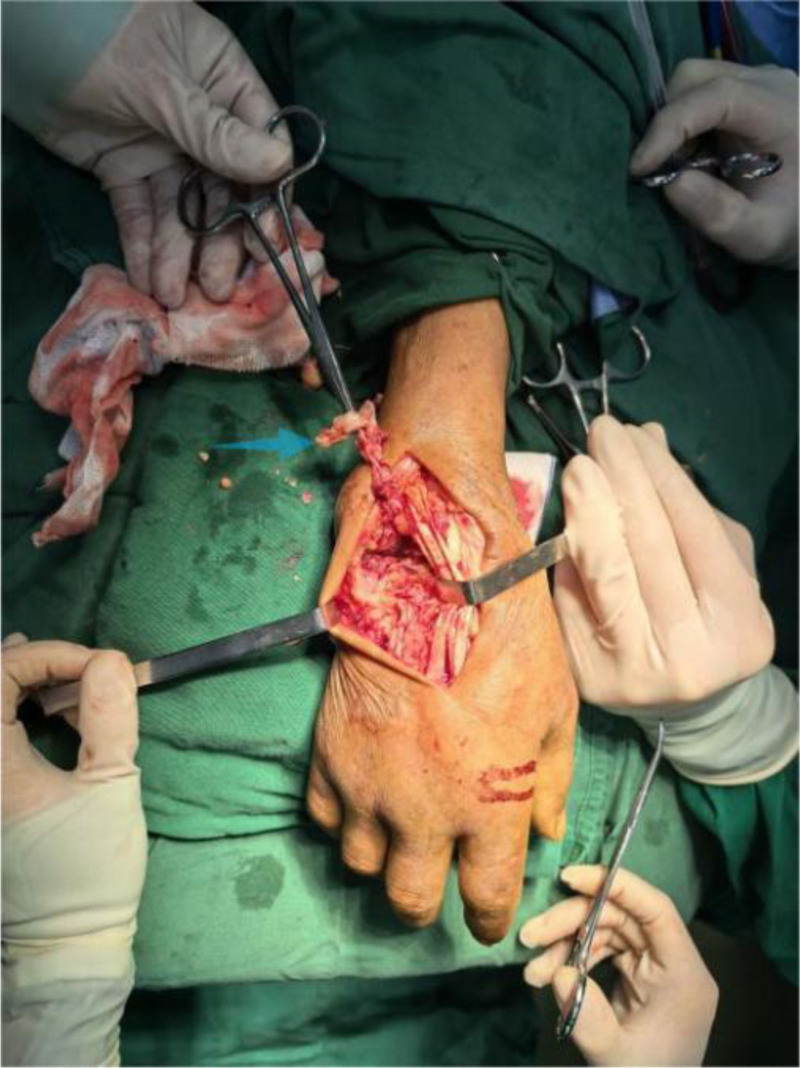
Blue arrow: rupture of the extensor carpi ulnaris and extensor digiti minimi tendons.

**Figure 4.**
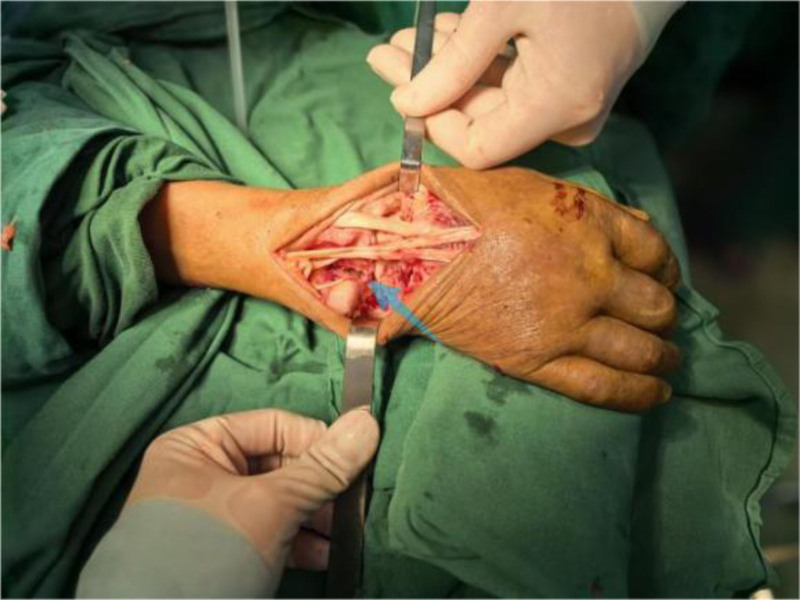
Blue arrow: dislocated distal radioulnar joint and eroded ulnotriquetral ligament.

Operative findings: arthroscopic examination revealed hyperemic synovium with nodular proliferation, notably devoid of hemosiderin deposition (Fig. 1). Diagnostic uncertainty prompted synovial biopsy for frozen section analysis and the collection of turbid joint fluid for bacteriological culture. Frozen pathology revealed diffusely proliferating synoviocytes and multinucleated giant cells, exhibiting mild-to-moderate atypia (Fig. 2). Discrepancies between the preoperative diagnosis and intraoperative findings necessitated conversion to open surgery via combined volar and dorsal midline approach. Exploration revealed extensive caseous necrotic material and granulomatous tissue within the flexor tendon sheaths and extensor carpi ulnaris (ECU) sheaths. Complete tendon ruptures were observed in the extensor digiti minimi (EDM) and ECU at the ulnar styloid level (Fig. 3). The DRUJ exhibited marked dissociation with ligamentous insufficiency (Fig. 4). While the articular cartilage of the carpal bones and distal radius appeared intact, the triquetrum demonstrated pathological fragility with a preserved trabecular architecture. Radical debridement was performed, followed by the repair of the ruptured EDM and ECU tendons. Primary arthrodesis was deferred because of a suspected mycobacterial infection. Postoperative stabilization was achieved with plaster immobilization of the wrist and DRUJ with staged arthrodesis planned following antitubercular therapy. All excised tissues were subjected to histopathological analysis.

### 2.3. Definitive histopathology

**Figure 5.**
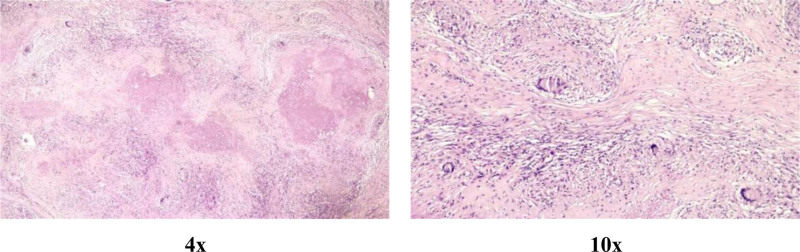
H&E-stained postoperative paraffin-embedded sections: reveals an area of caseous necrosis. H&E = hematoxylin and eosin.

Paraffin sections revealed granulomatous synovitis with extensive caseous necrosis (Fig. 5). Special stains were negative for acid-fast bacilli (AFB) and fungal elements (Periodic acid-Schiff). These features confirmed tuberculous granulomatous inflammation. Bacteriological cultures showed no growth after standard incubation.

## 3. Discussion

*Mycobacterium tuberculosis* typically infects the lungs and causes pulmonary disease. In contrast, infections of the skeletal muscle system most commonly involve the vertebrae. Tuberculous granulomatous tenosynovitis affecting the wrist joint is clinically rare, accounting for <1% of all osteoarticular TB cases.^[7,8]^ Hematogenous dissemination is the predominant route of infection. Predisposing factors include immunosuppression, immunocompromised status, malnutrition, trauma, joint overuse, and advanced age.^[9]^ Although the exact pathogenesis in this case remains unclear, it has been postulated to be associated with hematogenous spread, malnutrition, and chronic overuse of the affected limb. Tuberculous tenosynovitis more frequently involves flexor tendon sheaths than extensor sheaths.^[10]^ Notably, this case exhibited simultaneous involvement of the wrist joint and extensor and flexor tendons.

**Figure 6.**
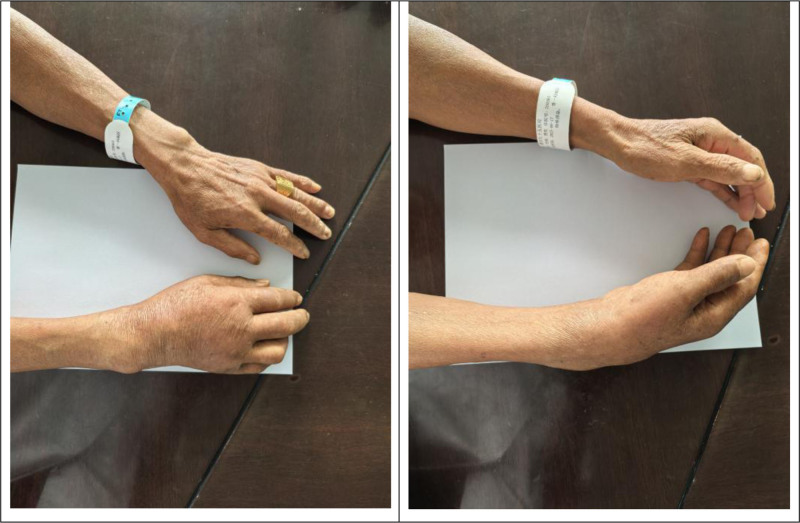
Preoperative appearance of the right wrist (dorsal and lateral views): prominence over the ulnar styloid region with diffuse swelling.

Musculoskeletal TB often presents a significant diagnostic challenge owing to its insidious onset and nonspecific symptoms, typically resulting in a diagnostic delay of 16 to 19 months.^[11]^ Such delays can lead to impaired wrist function and severe complications.^[12]^ In the present case, a definitive diagnosis was established within 2 weeks of the initial outpatient visit, following the return of pathological results. Providing a reliable basis for subsequent anti-tuberculous therapy. However, obtaining an accurate diagnosis is difficult. This patient exhibited an atypical presentation of wrist joint and tendon sheath TB, which lacked classic systemic symptoms. Symptoms were limited to chronic pain, diffuse swelling (Fig. 6), and functional limitations, closely mimicking conditions such as PVNS, rheumatoid arthritis, and giant cell tumor of the tendon sheath.

**Figure 7.**
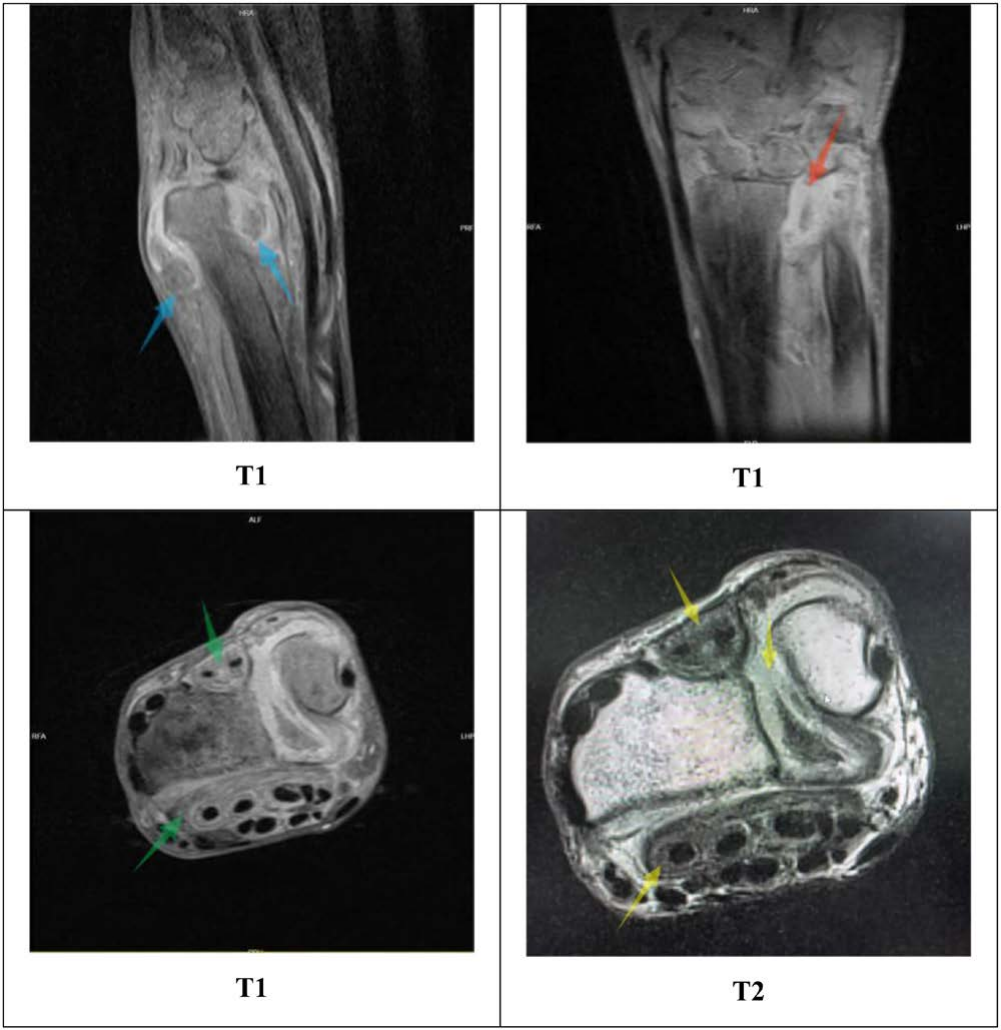
Preoperative wrist MRI (blue arrow): T1-weighted sagittal image: diffuse low-signal synovium with nodular changes, no significant bone marrow edema. T1-weighted coronal image (red arrow): nodular intermediate-to-low signal intensity between the distal radius and ulna indicating significant radioulnar joint incongruity. T1-weighted axial image (green arrow): extensive diffuse low signal intensity within the synovium and sheaths of the extensor and flexor tendons. T2-weighted axial image (yellow arrow): low signal intensity within the flexor and extensor tendon sheaths, suggesting hemosiderin deposition. MRI = magnetic resonance imaging.

**Figure 8.**
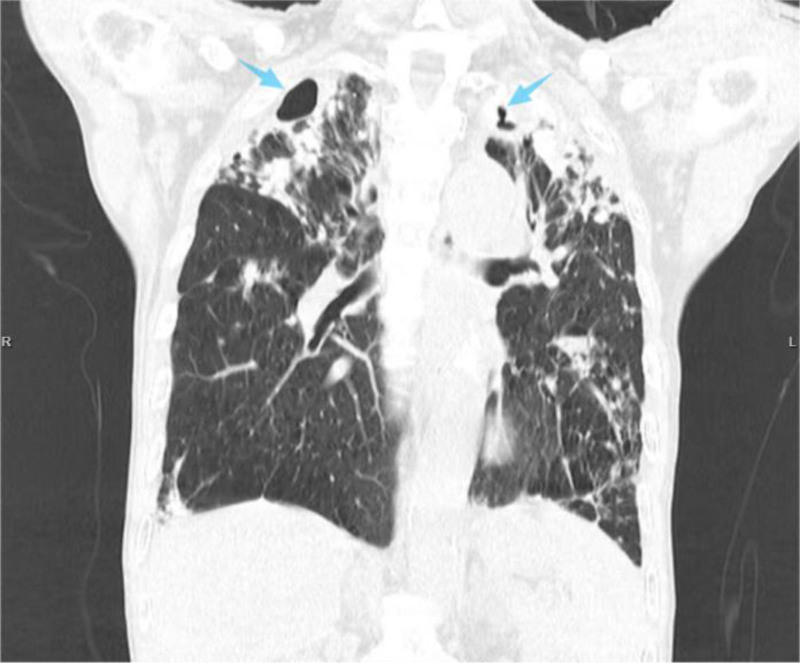
Preoperative chest CT: scans demonstrating nodules, masses, cavitary lesions (blue arrow), and fibrotic changes in the bilateral upper lung lobes. CT = computed tomography.

Preoperative investigations presented further diagnostic pitfalls. T-SPOT.TB results showed a nil control (N): 0.501 IU/mL, a positive control (P): >10 IU/mL, and a tuberculous antigen (T): 4.506 IU/mL. While partially suggestive of positivity, it is inconclusive for confirming TB, consistent with the documented suboptimal sensitivity of immunological assays for extrapulmonary TB and their potential for high false-positive rates.^[13]^ The preoperative C-reactive protein level was 5 mg/L and the ESR was 25 mm/h. Although ESR was elevated and there is a known association between serum biomarker levels and infection, both markers lack diagnostic specificity.^[14,15]^ Repeated sputum AFB smears were negative, and imaging findings further contributed to misdiagnosis. Wrist magnetic resonance imaging revealed diffuse synovial thickening and enhancement with low signal intensity on T2-weighted images, suggestive of hemosiderin deposition, with features highly overlapping with PVNS (Fig. 7). This constituted another key factor in the preoperative misdiagnosis. Chest CT demonstrated nodules, masses, cavities, and fibrotic changes (Fig. 8). Given the patient’s occupation as a miner, pneumoconiosis was considered as the primary diagnosis. However, pulmonary cavities can arise from various etiologies, including malignancy, TB, fungal infection, and pneumoconiosis.^[16]^ Multidisciplinary consultations (radiology, pulmonology) could not definitively establish whether the lung lesions represented active TB, pneumoconiosis, or a superimposed infection, recommending sputum culture for confirmation, which subsequently yielded negative results on multiple attempts.

The uncertainty surrounding the preoperative diagnosis prompted the decision to proceed with arthroscopic exploration and intraoperative frozen section analysis.

Regarding tuberculous tenosynovitis and osteoarticular TB, a characteristic intraoperative finding is the presence of rice bodies.^[8,17,18]^ In contrast, PVNS typically exhibits reddish-brown villonodular synovium and hemosiderin deposition on arthroscopy.^[19]^ During this procedure, the absence of classic hemosiderin deposition(Fig. 1), coupled with the observation of suspicious rice bodies and turbid joint fluid, raised the initial suspicion. However, significant joint destruction was absent arthroscopically, and intraoperative frozen section analysis revealed only synovial-like cells, giant cells, and mild-to-moderate cellular atypia, failing to identify caseous necrosis or characteristic granuloma.

Consequently, these findings led to an initial misdiagnosis of PVNS, which led to a diagnostic dilemma.

**Figure 9.**
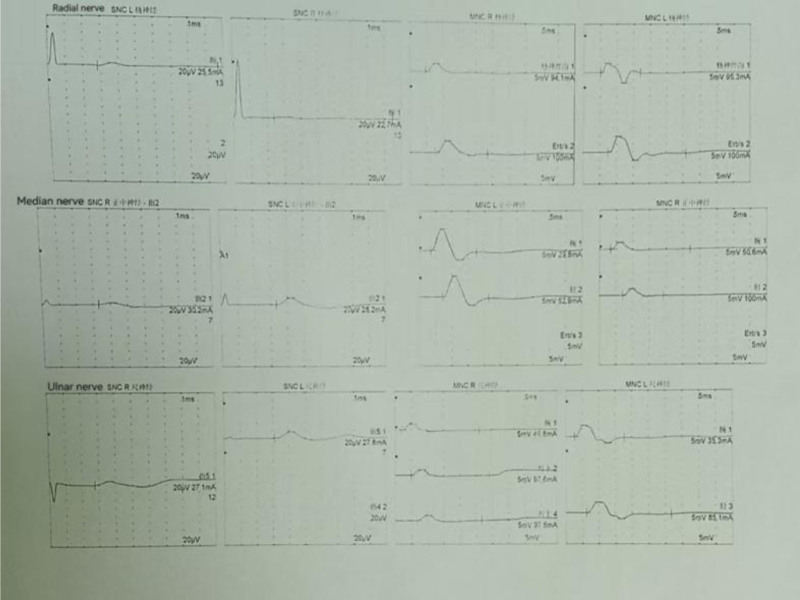
Nerve waveforms: motor nerve conduction studies (CMAPs) revealed reduced distal amplitudes in the median, ulnar, and radial nerves. Sensory nerve conduction studies (SNAPs) showed reduced distal amplitudes in the median, ulnar, and radial nerves, with conduction velocities largely preserved compared with the contralateral side. These findings suggested the possibility of nerve compression. CMAP = compound muscle action potentials, SNAP = sensory nerve action potentials.

**Figure 10.**
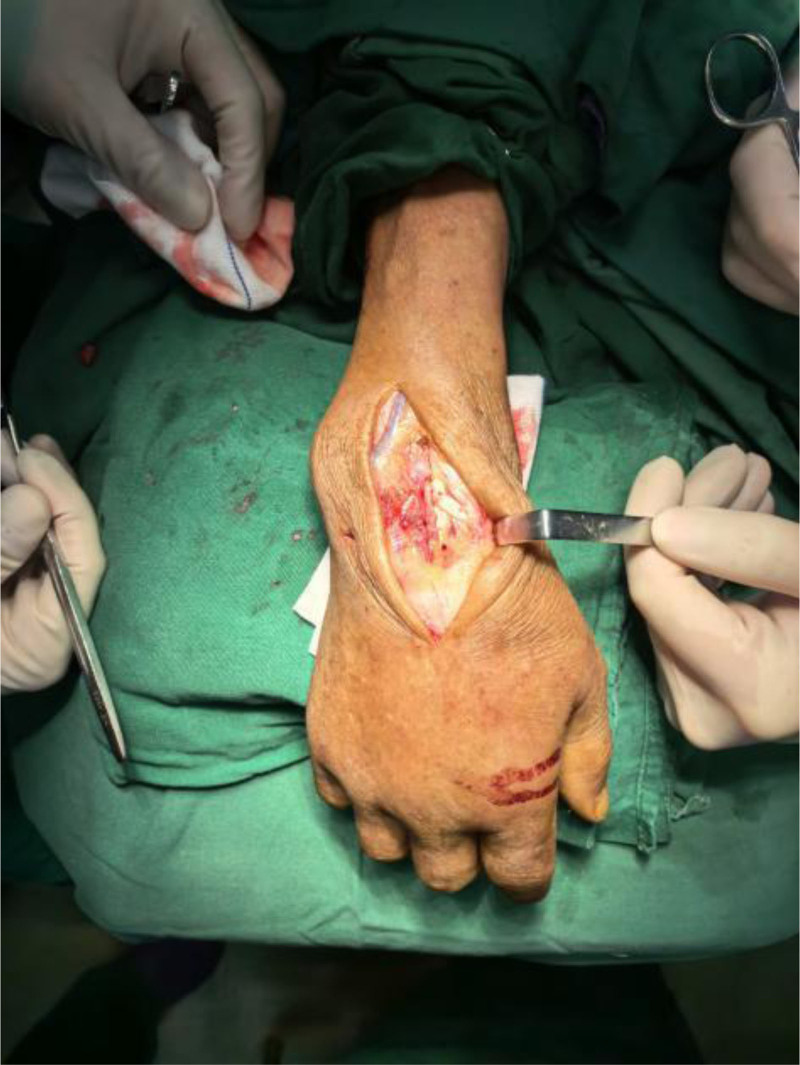
Intraoperative open view: grayish-yellow granulomatous tissue within the extensor tendon sheath, covering a wide area.

Given the preoperative presentation, including wrist pain, nerve compression (Fig. 9), joint instability, restricted mobility, deformity, and extensive lesion involvement (Figs. 6 and 10), radical debridement via open surgery was deemed necessary regardless of the underlying cause (infectious or otherwise).^[12]^ However, the appropriateness of primary wrist arthrodesis remains debatable.

Further considerations influencing the diagnostic reassessment included:

Intraoperative arthroscopic findings: the observation of suspicious rice bodies and pus.

Preoperative chest CT: revealing cavitary lesions. While multiple pathologies can cause cavities, pulmonary TB is a common etiology.^[20,21]^ Pneumoconiosis typically presents on CT as cavities, nodules, fibrotic pleural changes, and emphysema, often exhibiting homogeneous morphology and symmetric distribution. In contrast, pneumoconiosis complicated by TB infection demonstrates a diverse pattern, including nodules, patches, consolidation, fibrosis, and cavities, frequently with asymmetric or unilateral distribution.^[22-24]^

**Figure 11.**
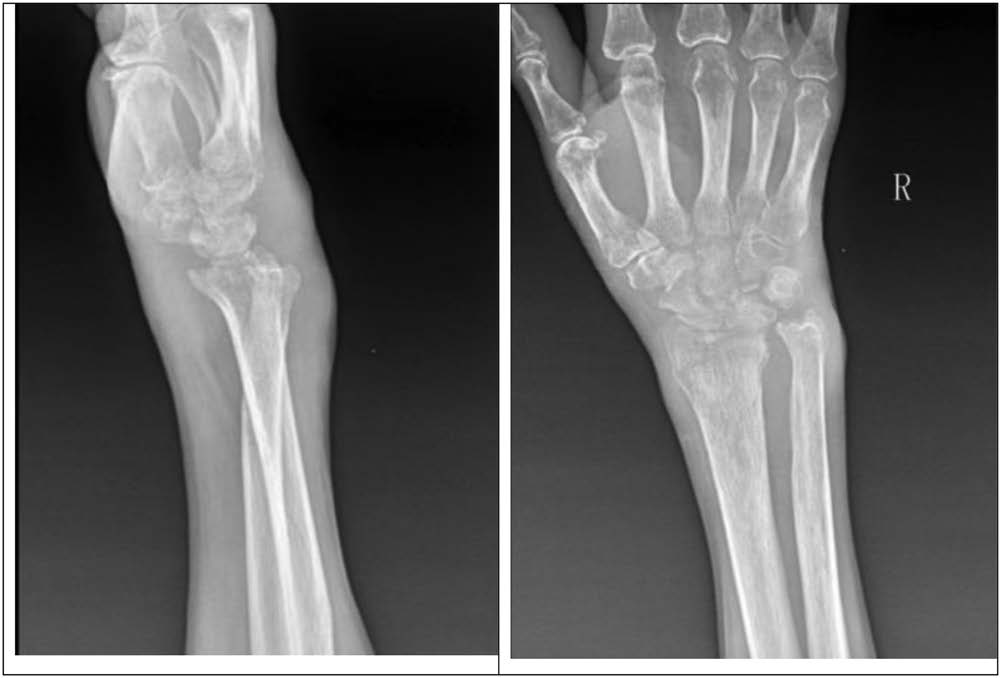
Outpatient wrist radiography: demonstration of joint space narrowing, osteoporosis, and bone destruction.

**Figure 12.**
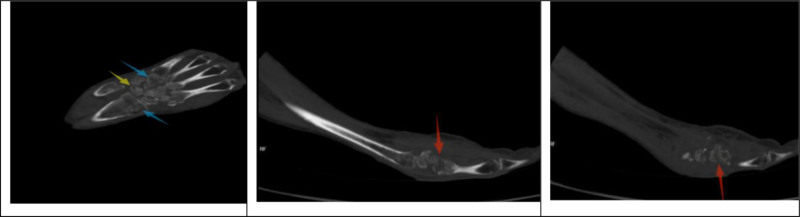
Preoperative wrist CT scan: reveal trabecular rarefaction and osteoporotic changes in the distal radius and ulna, carpal bones, and metacarpals (blue arrow). Lytic bone destruction with cystic cavities was evident in the lunate and triquetrum (yellow arrow). Erosive destruction and joint space narrowing were visible at the base of the second metacarpal and trapezoid-capitate joints (red arrow). No significant sclerotic margins are observed. CT = computed tomography.

Preoperative radiography revealed significant bone destruction (Figs. 11 and 12). Tuberculous destruction is typically more pronounced than in PVNS, manifesting as cystic changes, a moth-eaten appearance, and sclerotic margins around destructive areas.^[25]^

Preoperative physical and imaging signs: a prominent “piano-key sign” at the DRUJ, indicating instability, corroborated by imaging evidence of DRUJ dislocation. This suggested ligamentous and capsular destruction. TB is known to erode ligaments, potentially leading to rupture.^[26]^

Post-arthroscopy, the diagnosis leaned more towards tuberculous tenosynovitis/arthritis. While anti-TB chemotherapy is the mainstay of treatment for TB, the role of adjunctive surgery requires careful consideration, as it can aid in preserving joint function.^[27]^ Considering the constellation of findings – extensive lesions, instability, nerve compression, and tendon pathology – it was determined that open radical debridement was necessary and beneficial for functional recovery. However, arthrodesis was deferred at a later stage following anti-TB treatment.

Critical intraoperative findings during open surgery confirmed the suspicion that tuberculous tenosynovitis can cause complete tendon rupture.^[28,29]^ Ruptures of the EDM and ECU tendons were intraoperatively observed. While PVNS commonly involves tendon sheaths, complete rupture is relatively uncommon, and marked separation of the DRUJ is evident. PVNS rarely causes severe instability, whereas tuberculous ligament destruction is typically more aggressive.^[26,28]^ These features significantly exceeded the typical presentation of PVNS, prompting extensive debridement and submission of all excised tissues for comprehensive histopathological examination of permanent sections. The diagnosis was ultimately confirmed by the identification of caseous necrosis. The initial error in the intraoperative frozen section diagnosis was attributed to limitations inherent to the technique and, crucially, insufficient communication of the patient’s pulmonary findings (cavitary lesions) to the pathologist. Definitive diagnosis relies on conventional histopathology; the identification of caseous necrosis, positive AFB staining, or positive *M tuberculosis* PCR constitutes the diagnostic gold standard.^[30-32]^ Conversely, PVNS is characterized histologically by synovial cell hyperplasia, abundant hemosiderin deposition, multinucleated giant cells, and foam cells without necrosis or granulomas.^[33]^ For patients with negative serological tests, atypical imaging findings, unexplained bone destruction, and suspicious pulmonary lesions, clinicians should maintain a high index of suspicion for TB even if initial pathological examinations (such as intraoperative frozen section) support an alternative diagnosis.

**Figure 13.**
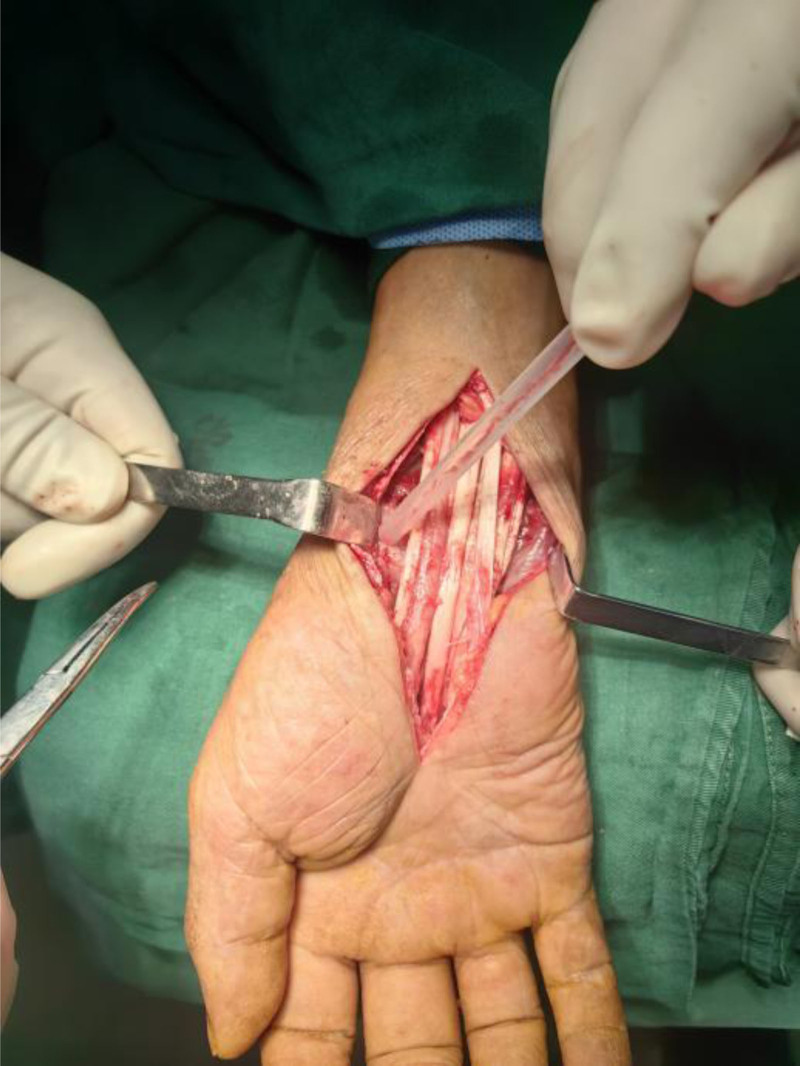
Flexor tendons following debridement of the lesion.

**Figure 14.**
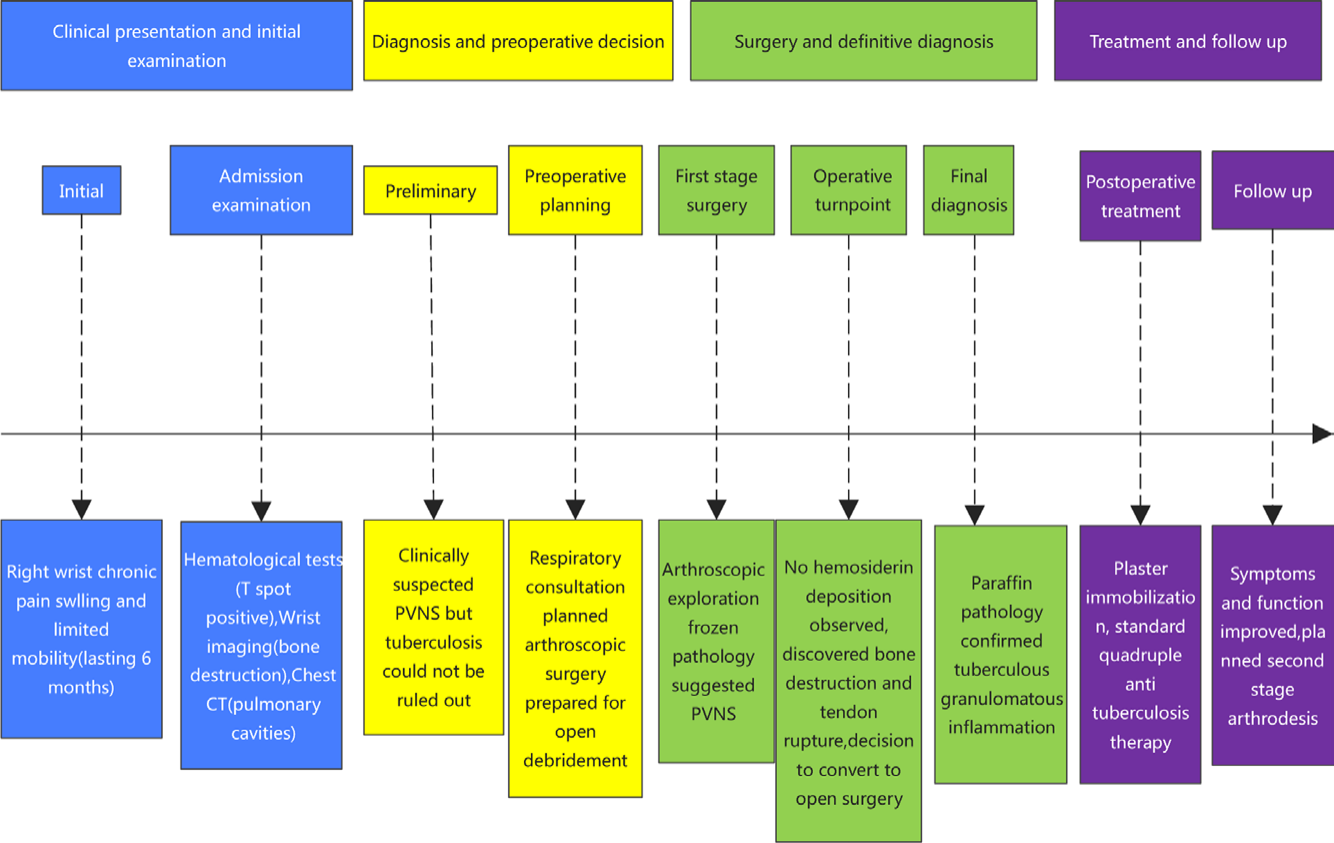
Timeline of diagnosis and treatment for wrist tuberculous granulomatous inflammation.

The management of wrist joint TB prioritizes anti-tuberculous chemotherapy, with surgical intervention serving an adjunctive role. In treatment decision-making, surgical indications require careful consideration,^[34]^ and standard quadruple anti-tuberculous therapy (isoniazid, rifampicin, pyrazinamide, ethambutol) is the cornerstone for confirmed TB. If a case cannot be diagnosed clearly, surgical indications include diagnostic biopsy, debridement of the lesions, decompression of nerves, vessels, or tendons. Repair of the tendon rupture. Stabilization of an unstable or arthritic joint. Correction of deformity: anti-tuberculous therapy is mandatory postoperatively once the diagnosis is pathologically confirmed. In this case, although the diagnosis remained elusive preoperatively and intraoperatively, debridement, nerve decompression, and tendon repair were successfully performed (Fig. 13). Given the suspected tuberculous etiology, primary arthrodesis was avoided, and postoperative stabilization was achieved with cast immobilization, creating favorable conditions for subsequent anti-tuberculous therapy and delayed arthrodesis. Definitive wrist arthrodesis was planned as a second-stage procedure following chemotherapy, and open surgery proved more effective for managing this patient’s extensive pathology and structural damage, resulting in immediate postoperative symptomatic and functional improvement. At the 3-month postoperative follow-up, the patient’s pain had resolved, and no further oral analgesics were required. With the forearm in neutral zero position, the range of motion of the right forearm was 30° of pronation and 15° of supination. The metacarpophalangeal joint of the right hand had a 5° range of motion, indicating some improvement compared with the preoperative status, albeit limited (Fig. 14).

## 4. Conclusion

This case highlights that TB of the wrist joint and tendon sheath must remain a critical differential diagnosis in patients presenting with wrist swelling, pain, and bony destruction, even in the absence of systemic symptoms, despite negative TB-specific investigations. Preoperative imaging demonstrated significant overlap with PVNS, relying solely on radiological findings prone to misdiagnosis. Intraoperative frozen section pathology has inherent limitations. Furthermore, key intraoperative arthroscopic findings specifically, the absence of typical hemosiderin deposition, presence of suspicious rice body-like formations, marked preoperative bony destruction, and joint instability exceeded the typical spectrum of PVNS manifestations. These findings necessitated a high index of suspicion, prompting a transition to an open surgical approach. This allowed thorough debridement and adequate tissue sampling for pathological analysis to obtain a definitive diagnosis, which was ultimately established using paraffin section histopathology. Successful management of wrist joint and tendon sheath TB requires a combined approach of sufficient-course anti-TB chemotherapy and radical surgical debridement. Enhance awareness of rare diseases, maintain a high level of vigilance, thoroughly integrate preoperative and intraoperative findings, actively pursue multidisciplinary consultations, and ensure effective communication with auxiliary departments are essential to avoid delays in diagnosis and treatment.

## Author contributions

**Conceptualization:** Yuchao Lin.

**Methodology:** Yuchao Lin, Junlin Liu, Weilong Lin.

**Supervision:** Yuchao Lin.

**Validation:** Yudong Huang, Xiaohua Zheng.

**Writing – original draft:** Yuchao Lin, Junlin Liu.

**Writing – review & editing:** Yuchao Lin, Junlin Liu, Weilong Lin.

## References

[1] Onah CK Azuogu BN Ossai EN. Addressing constraints to informal providers’ involvement in tuberculosis control: a qualitative study of patent medicine dealers and tuberculosis programme managers. Glob Health Res Policy. 2021;6:43. 34743759 10.1186/s41256-021-00227-x PMC8574043

[2] GBD. Burden of 375 diseases and injuries, risk-attributable burden of 88 risk factors, and healthy life expectancy in 204 countries and territories, including 660 subnational locations, 1990-2023: a systematic analysis for the Global Burden of Disease Study 2023. Lancet. 2025;406:1873–922. 41092926 10.1016/S0140-6736(25)01637-X PMC12535840

[3] Sbai MA Benzarti S Boussen M Maalla R. Tuberculous flexor tenosynovitis of the hand. Int J Mycobacteriol. 2015;4:347–9. 26964820 10.1016/j.ijmyco.2015.06.003

[4] Ngoc CT Tuan NC Thinh NP Duc NM. Clinical characteristics and treatment outcomes of tuberculous tenosynovitis of the hand and wrist. Int J Med Sci. 2023;20:985–92. 37324187 10.7150/ijms.84727 PMC10266042

[5] El Aissaoui T Tebbaa El Hassali A Lachkar A Yacoubi H Abdeljaouad N. A rare manifestation of extrapulmonary tuberculosis: tenosynovitis of wrist and multiple fingers. Cureus. 2025;17:e84025. 40510107 10.7759/cureus.84025 PMC12161165

[6] Teo SC George J Kamarul T. Tubercular synovitis mimicking rheumatoid nodules. Med J Malaysia. 2008;63:159–61. 18942309

[7] Higuchi S Ishihara S Kobayashi H Arai T. A mass lesion of the wrist: a rare manifestation of tuberculosis. Intern Med. 2008;47:313–6. 18277037 10.2169/internalmedicine.47.0495

[8] Bayram S Erşen A Altan M Durmaz H. Tuberculosis tenosynovitis with multiple rice bodies of the flexor tendons in the wrist: a case report. Int J Surg Case Rep. 2016;27:129–32. 27611797 10.1016/j.ijscr.2016.08.021 PMC5018083

[9] Lall H Nag SK Jain VK Khare R Mittal D. Tuberculous extensor tenosynovitis of the wrist with extensor pollicis longus rupture: a case report. J Med Case Rep. 2009;3:142. 20062777 10.1186/1752-1947-3-142 PMC2803811

[10] Tian Y Zhou HB Yi K Wang KJ. Idiopathic tenosynovitis of the wrist with multiple rice bodies: a case report and review of literature. World J Clin Cases. 2022;10:11908–20. 36405290 10.12998/wjcc.v10.i32.11908 PMC9669876

[11] Yao DC Sartoris DJ. Musculoskeletal tuberculosis. Radiol Clin North Am. 1995;33:679–89. 7610238

[12] Singh M Jeyaraman M Jeyaraman N Jayakumar T Iyengar KP Jain VK. *Mycobacterium tuberculosis* infection of the wrist joint: a current concepts review. J Clin Orthop Trauma. 2023;44:102257. 37841656 10.1016/j.jcot.2023.102257 PMC10568419

[13] Chen H Nakagawa A Takamori M. Diagnostic accuracy of the interferon-gamma release assay in acquired immunodeficiency syndrome patients with suspected tuberculosis infection: a meta-analysis. Infection. 2022;50:597–606. 35249210 10.1007/s15010-022-01789-9 PMC9151521

[14] Moein SA Fereidooni R Niakan R Kousari A. *Mycobacterium tuberculosis*-induced multiple tenosynovial masses with rice bodies: a case report. Clin Case Rep. 2023;11:e8228. 38125627 10.1002/ccr3.8228 PMC10731109

[15] Moldovan F. Role of serum biomarkers in differentiating periprosthetic joint infections from aseptic failures after total hip arthroplasties. J Clin Med. 2024;13:5716. 39407776 10.3390/jcm13195716 PMC11476511

[16] Ryu JH Swensen SJ. Cystic and cavitary lung diseases: focal and diffuse. Mayo Clin Proc. 2003;78:744–52. 12934786 10.4065/78.6.744

[17] Ergun T Lakadamyali H Aydin O. Multiple rice body formation accompanying the chronic non-specific tenosynovitis of flexor tendons of the wrist. Radiat Med. 2008;26:545–8. 19030963 10.1007/s11604-008-0270-7

[18] Korkmaz MC Tolu S Şimşek S. A rare case of flexor tenosynovitis due to tuberculosis in hand and wrist: a case report. Acta Chir Orthop Traumatol Cech. 2021;88:237–9. 34228622

[19] Poutoglidou F Metaxiotis D Mpeletsiotis A. Pigmented villonodular synovitis of the knee joint in a 10-year-old patient treated with an all-arthroscopic synovectomy: a case report. Cureus. 2020;12:e11929. 33425510 10.7759/cureus.11929 PMC7785498

[20] Dong ZP Cui QY Pan SP Zhao YX. Clinical characteristics and follow-up analysis of 63 cases of silicosis complicated with cavity-pulmonary tuberculosis. Zhonghua Lao Dong Wei Sheng Zhi Ye Bing Za Zhi. 2024;42:268–70. 38677989 10.3760/cma.j.cn121094-20230329-000103

[21] Hunter RL. Tuberculosis as a three-act play: a new paradigm for the pathogenesis of pulmonary tuberculosis. Tuberculosis (Edinb). 2016;97:8–17. 26980490 10.1016/j.tube.2015.11.010 PMC4795183

[22] Baur X Sanyal S Abraham JL. Mixed-dust pneumoconiosis: review of diagnostic and classification problems with presentation of a work-related case. Sci Total Environ. 2019;652:413–21. 30368172 10.1016/j.scitotenv.2018.10.083

[23] Cai ZC Wang SS Chen YX. Image features and clinical significance of pneumoconiosis with large shadow. Zhonghua Lao Dong Wei Sheng Zhi Ye Bing Za Zhi. 2016;34:214–7. 27220446 10.3760/cma.j.issn.1001-9391.2016.03.015

[24] Elkard I Zaghba N Benjelloun H Bakhatar A Yassine N. La silicotuberculose. Rev Pneumol Clin. 2016;72:179–83. 26790716 10.1016/j.pneumo.2015.10.003

[25] Marais LC Nieuwoudt L Nansook A Menon A Benito N. Tuberculous arthritis of native joints - a systematic review and European Bone and Joint Infection Society workgroup report. J Bone Jt Infect. 2023;8:189–207. 37780528 10.5194/jbji-8-189-2023 PMC10539782

[26] Baidoo PK Baddoo D Ocloo A Agbley D Lartey S Baddoo NA. Tuberculous tenosynovitis of the flexor tendons of the wrist: a case report. BMC Res Notes. 2018;11:238. 29636100 10.1186/s13104-018-3343-4 PMC5894222

[27] Manske J Tille E Schlüßler A Biewener A Nowotny J. Tuberculosis of the elbow joint: the complexity of diagnosis and treatment-A case report and review of literature. J Med Case Rep. 2025;19:88. 40025616 10.1186/s13256-025-05102-8 PMC11874434

[28] Takahashi M Hirano T Kondo K Mitsuhashi T. Tuberculous flexor tenosynovitis around the wrist causing massive tendon disruption: a case report. Modern Rheumatol Case Rep. 2019;3:108–13.

[29] Lee SM Lee WJ Song AR. Tuberculous tenosynovitis and ulnar bursitis of the wrist. Ann Rehabil Med. 2013;37:572–6. 24020040 10.5535/arm.2013.37.4.572 PMC3764354

[30] Akyildiz EU. Intraoperative pathology consultation for pulmonary lesions: errors and deferrals. Int J Clin Exp Pathol. 2015;8:7961–6. 26339362 PMC4555690

[31] Sams SB Wisell JA. Discordance between intraoperative consultation by frozen section and final diagnosis. Int J Surg Pathol. 2017;25:41–50. 27507675 10.1177/1066896916662152

[32] Roy S Parwani AV Dhir R Yousem SA Kelly SM Pantanowitz L. Frozen section diagnosis: is there discordance between what pathologists say and what surgeons hear? Am J Clin Pathol. 2013;140:363–9. 23955455 10.1309/AJCPHUE5ENZDU4DJ

[33] Giri Goswami DS Bhake DA Khan DS Agrawal DS Bhawani DJ. Case Report: cytodiagnosis of pigmented villonodular synovitis involving carpal bones of right wrist. F1000Res. 2023;12:1490. 38957200 10.12688/f1000research.141797.1 PMC11217717

[34] Kabakaş F Uğurlar M Turan DB Yeşiloğlu N Mersa B Özçelik IB. Flexor tenosynovitis due to tuberculosis in hand and wrist: is tenosynovectomy imperative? Ann Plast Surg. 2016;77:169–72. 26418769 10.1097/SAP.0000000000000603

